# Disordered Flanks Prevent Peptide Aggregation

**DOI:** 10.1371/journal.pcbi.1000241

**Published:** 2008-12-19

**Authors:** Sanne Abeln, Daan Frenkel

**Affiliations:** FOM Institute for Atomic and Molecular Physics, Amsterdam, The Netherlands; Columbia University, United States of America

## Abstract

Natively unstructured or disordered regions appear to be abundant in eukaryotic proteins. Many such regions have been found alongside small linear binding motifs. We report a Monte Carlo study that aims to elucidate the role of disordered regions adjacent to such binding motifs. The coarse-grained simulations show that small hydrophobic peptides without disordered flanks tend to aggregate under conditions where peptides embedded in unstructured peptide sequences are stable as monomers or as part of small micelle-like clusters. Surprisingly, the binding free energy of the motif is barely decreased by the presence of disordered flanking regions, although it is sensitive to the loss of entropy of the motif itself upon binding. This latter effect allows for reversible binding of the signalling motif to the substrate. The work provides insights into a mechanism that prevents the aggregation of signalling peptides, distinct from the general mechanism of protein folding, and provides a testable hypothesis to explain the abundance of disordered regions in proteins.

## Author Summary

In their natural cellular environment proteins are dissolved in a concentrated aqueous solution of biomolecules. Even under such crowded conditions, proteins must not clump together or aggregate; otherwise their biological functions may be compromised, and the cell could die. Diseases such as Parkinson and Alzheimer are thought to be caused by aggregation of specific proteins. Evolutionary pressure generally ensures that proteins do not aggregate in their natural biochemical environment. A well-known mechanism to prevent aggregation is the folding of proteins, where the hydrophobic (attractive) part of the protein is buried inside the protein. Here we report a different mechanism that can prevent the aggregation of proteins. Recently, it was discovered that many proteins contain regions that are disordered (not folded) in their natural environment. We show with coarse-grained simulations that aggregation of small hydrophobic binding motifs can be prevented by embedding the motifs in disordered regions: the disordered regions of different proteins obstruct or sterically hinder the formation of aggregates. Moreover, our simulations show that the disordered regions have no adverse effect on the biological function of the binding motifs, because they do not obstruct the binding and folding of the binding motif on its specific substrate.

## Introduction

The biological function of many proteins is determined by their native, three-dimensional structure and unfolded (or incorrectly folded) copies of such proteins tend to be inactive, if not outright dangerous.

However, many proteins contain large regions (>30 amino acids) that are disordered in their natural physico-chemical environment [1]–[4]; some proteins are even entirely disordered [5],[6]. As more peptide sequences are being studied, it is becoming increasingly clear that natively-disordered sequences are far more common than previously thought. Disordered sequences have been found on a large number of eukaryotic genes (>30%) [2],[5],[7],[8]. Moreover, the number of genes on a genome with disordered regions appears to increase with the complexity of the species [2],[5],[7],[8].

Despite a lack of stable structure in the native form of the protein, disorder is strongly associated with specific cellular functions, most significantly with cell signalling and regulatory processes [9]–[14]. Several suggestions have been made about the possible benefits of disordered regions in a protein: they could be more malleable, have a large binding surface, bind to diverse ligands, bind with high specificity and make the binding process reversible [1],[12],[15],[16]. Indeed, there exist numerous examples of natively disordered proteins that form a more defined structure upon binding to a ligand [17], implying that the protein loses conformational entropy on binding.

Disordered regions (peptide sequences that are generally unfolded) and natively unstructured binding regions (sequences that only take a specific structure upon binding) have some general features. Disordered regions contain fewer hydrophobic, more hydrophilic, more charged amino acids and more repeats in their sequence as compared to natively structured proteins [6].

**Figure 1:**
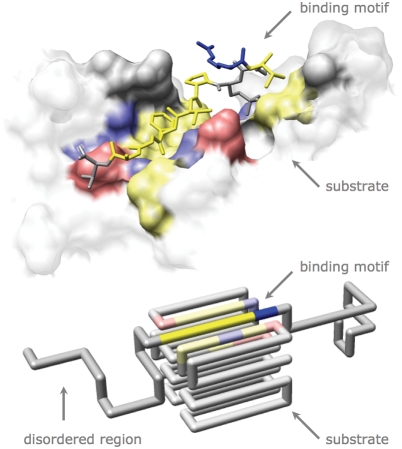
Linear binding motifs. Top: Example of a linear binding motif bound to its substrate. CtIP phosphopeptide is bound to BRCT repeats of BRCA1 (1Y98). Bottom: Model of a binding motif. The motif, with sequence RWWLY, is designed to bind specifically to the substrate. The yellow residues are hydrophobic, the blue negatively charged, the red positively charged and the grey hydrophilic.

On the other hand interfacial regions between a natively unstructured binding region and a rigid protein contain relatively more hydrophobic and fewer charged contacts, as compared to rigid-rigid interfaces [18]. In general, only a small (hydrophobic) motif of the disordered region is involved in the actual binding and this binding motif remains in an extended configuration even upon binding and ‘folding’ [19]–[21]. As a consequence, the exposed binding area per residue is relatively large [15],[18] (see Figure 1).

Recent studies have revealed that many small (linear) binding motifs are surrounded by disordered regions [22],[23]. A typical linear binding motif contains some 6 residues and is surrounded by approximately 20 residues that are natively unstructured [23]. The binding motifs are typically more hydrophobic than the flanking residues. Since the binding regions are relatively small, they are unlikely to form fully folded (or specific) structures in solution when not bound to a substrate. In this study we focus on the steric effects of the disordered regions adjacent to small hydrophobic binding motifs.

As the presence of disordered regions near small binding motifs appears to be generic, it seems justified to use a generic model. The nature of the coarse-grained model allows us to simulate the specificity, steric hindrance, configurational and translational entropy of the peptide chain. Each residue of the peptide chain occupies a single point on a cubic lattice. The lattice makes efficient movements in the peptide chain possible so that many different configurations of the chain can be sampled with a Monte Carlo algorithm. Residues on neighbouring lattice points interact in a pairwise manner. Each of the 20 amino acids has a specific interaction energy with each of the other amino acids [24],[25]. For example, two neighbouring hydrophobic amino acids lower the internal energy and are thus attracted to each other. The large number of possible interactions and sequences enables the design of amino acid sequences that fold into a specific structure [26],[27]. Using these designed peptide sequences it is possible to describe the folding mechanism of highly specific folding [26],[27] or binding [16],[28]. However, due to its coarse-grained nature, the model would be unsuited to represent the structure or binding site of a specific, naturally occurring protein.

We use this coarse-grained model to investigate how the binding free energy of a short binding motif depends upon its structural environment: we simulate binding to a substrate for a flexible binding motif, a flexible motif embedded in an unstructured chain and a rigid binding motif embedded in a rigid structure (see Figure S1). The model of the substrate and binding region embedded in disordered flanks have been designed to contain the general features associated with disordered regions and natively unstructured binding regions, viz. an extended binding conformation, a large binding surface, hydrophobicity of the binding region and hydrophilic flanks.

We find that the binding motif embedded in a rigid structure unbinds at higher temperatures than either the flexible binding motif or the binding motif in a longer disordered region. The latter two binding free energies are very similar over the range of temperatures simulated. However, we show that even at low concentrations the (hydrophobic) binding motif aggregates with itself, and that the (hydrophilic) disordered flanks prevent such aggregation at temperatures relevant for reversible binding.

## Results

### Folding and Binding of Binding Motifs

To investigate how the binding free energy of a short binding motif depends upon its structural environment, a binding motif was designed to specifically bind in a groove of a rigid substrate (Figure 1). The amino acid sequence (Arg, Trp, Tr, Leu, Tyr) of this motif is predominantly hydrophobic, but contains a single charged amino acid. In our coarse-grained model, neighbouring hydrophobic residues attract each other, whereas amino acids of the same charge repel each other.

The binding of this binding motif was simulated embedded in three different structures: as a single flexible binding motif (BM), as a single flexible binding motif with disordered flanks of 15 Threonine residues on each side (BM disorder) and embedded in a rigid structure of Threonine residues (BM rigid), see Figures S1 and S2. Threonine is a hydrophilic amino acid. In our model contacts involving Threonine do not contribute to the internal energy of the configuration so that the internal energy of the binding motif bound to the substrate is the same for all three structures (see Methods).

**Figure 2:**
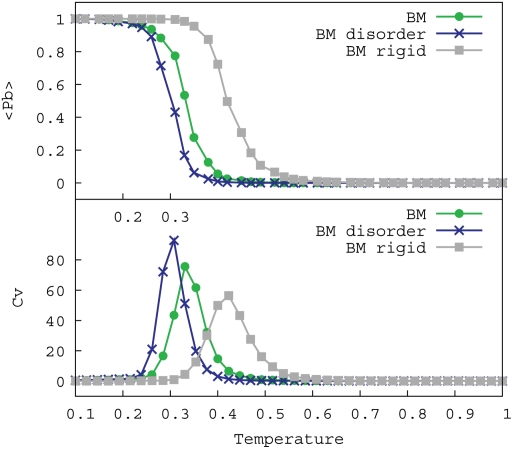
Reversible binding. Average amount of binding (〈*P_b_*〉, top) and heat capacity (*C_v_*, bottom) as a function of temperature shown for an isolated binding motif (BM), a binding motif within disordered flanks (BM disorder) and a rigid binding motif embedded in a rigid structure (BM rigid).

The binding and unbinding process was simulated at different temperatures, while the concentration of the substrate and peptide are kept constant. Figure 2 shows that at low temperatures (*T*<0.25) the average degree of binding (〈*P_b_*〉) is high, i.e. the binding motif is nearly always bound to the substrate, and at high temperatures (*T*>0.45) the average degree of binding is low. The flexible peptides (BM and BM disorder) are unstructured in the unbound state (see Figure S2).

There is a transition between the bound and unbound state at which reversible binding is possible. This transition can also be observed by the peak in the heat capacity (*C_v_*). Similar peaks in heat capacity are found at folding transitions of both simulated and real proteins (e.g., [29],[30]). The sharpness of the heat-capacity curve also indicates that the binding motif binds with high specificity to the substrate. Binding of an aspecific motif to the substrate would result in a much broader heat-capacity peak.

In nature binding motifs typically have a signalling function, implying that the peptide should be able to bind as well as unbind in the relevant temperature range. Figure 2 shows that the binding motif binds reversibly to the substrate for approximately 0.2<*T*<0.3.

Interestingly, Figure 2 shows that the disordered flanks have little effect on the binding free energy: the average amount of binding and heat capacity are similar over the entire temperature range for both flexible peptides (BM and BM disorder). Additional simulations showed that even with a much larger substrate the difference in binding free energy between the binding motif and the motif embedded in disordered flanks remains small. However, as previously reported [16], the flexibility of the binding motif itself lowers the difference in free energy between the bound and unbound state, since conformational entropy is lost upon binding to the substrate. Figure 2 shows that the temperature range for reversible binding of flexible peptide chains is lower than for a rigid binding motif.

### Aggregation of Small Binding Peptides

**Figure 3:**
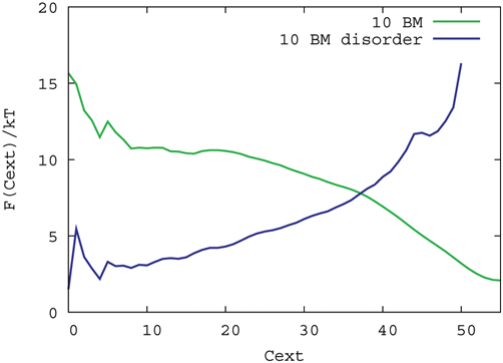
Aggregation free energy. Free energy as a function of external contacts at T = 0.23. The free energy is defined as *F*(*C_ext_*) = −*k_B_T* ln(*P*(*C_ext_*)) where *P*(*C_ext_*) is the probability of a configuration with *C_ext_* external contacts. The number of peptide chains was kept constant at 10. Free energies for 0<*C_ext_*<55 are displayed; free energies for a higher number of external contacts are dominated by finite size effects (10 peptides) effect of the system.

Even though disordered flanks appear to contribute little to the binding free energy, the collective contribution of many such flanks may be important. We simulated 10 binding motifs without the substrate to investigate the collective behaviour of the peptides. Figure 3 shows that 10 binding motifs without flanks tend to aggregate whereas those with flanks do not at a temperature at which reversible binding is possible; the lowest free energy configuration for 10 binding motifs with flanks is as free chains or in very small clusters, whereas the binding motifs without flanks make many more external contacts.

To investigate this phenomenon for a larger number of peptide chains, we simulated aggregation behaviour of the two types of binding motifs with a Grand Canonical Monte Carlo simulation, while keeping the free binding motifs at low concentration (see Methods).

**Figure 4:**
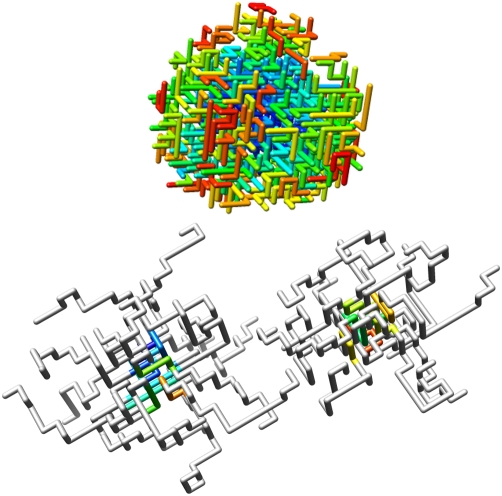
Aggregation of binding motifs. Top: snapshot of 301 aggregated binding motifs. Bottom: snapshot of two micelles formed by 18 binding motifs embedded in Threonine flanks (grey). The binding motifs have been given a colour ranging from blue to red according to their order of appearance in the simulation box.

**Figure 5:**
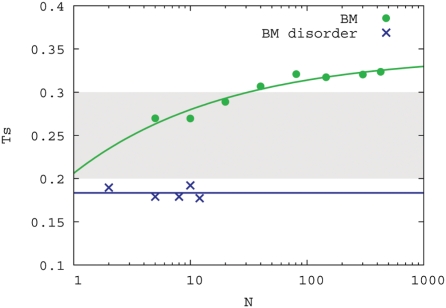
Melting temperatures of aggregated clusters. Cluster size (*N*) versus melting temperatures (*T_s_*) for different cluster sizes. The shaded area indicates the temperature range in which reversible binding is possible for the flexible binding motifs to the substrate (see Figure 2). Stable aggregates exist in the regions below the melting curves.

First, simulations starting from a single chain in the simulation box were performed at different temperatures. Many more external contacts form for the binding motif than for the binding motif embedded in disordered flanks (Figure 4). Moreover, the aggregates form at higher temperatures for binding motifs without disordered flanks. From these simulations we selected aggregates of different cluster sizes. Each cluster of aggregates was simulated at different temperatures to determine the transition temperature, *T_s_*, at which the aggregate would shrink rather than grow in size (Figure 5).

Comparing Figure 2 with Figure 5 it can be observed that the binding motifs (BM) are in an aggregated state at temperatures within the reversible binding regime, whereas the binding motifs with disordered (BM disorder) are fully dissolved. Figure 2 also shows that with increasing aggregate size the aggregates formed by binding motifs without disordered flanks become more difficult to melt, indicating that once an aggregate is formed it will be difficult to dissolve. Binding motifs embedded in disordered domains, generally form micelle-like structures that do not grow larger than approximately 12 chains (see Figure 4). Decreasing the length of the disordered flanks, down to 5 residues on each side of the binding motif, does not have a strong effect on the melting temperatures. In that case the micelles formed are somewhat larger.

The system also shows considerable hysteresis: the aggregated clusters melt at much higher temperatures than the ones at which they formed. Again, this effect is much smaller for binding motifs embedded in disordered flanks.

## Discussion

Our simulations suggest that the primary role of disordered flanks adjacent to small peptide binding motifs is to suppress aggregation in solution rather than to modify the binding strength to the substrate. This observation provides a rationale for the experimental observation that linear binding motifs are often found in disordered parts of a peptide chain [23].

In this work only a small difference in binding strength between binding motifs with and without disordered flanks is found. The model used here is based on the assumption that interactions between the disordered flanks and the substrate are of a steric nature. However our results do not preclude the possibility that the binding strength changes significantly if the disordered flanks have additional interactions with the substrate, for example through charged residues or a second binding motif. Our work focuses on the physical effect of disordered flanks that have no specific interaction with the substrate.

The isolated binding motifs described in the present paper would aggregate due to hydrophobic interactions. We suggest that such motifs, without hydrophilic flanks, are toxic. There is indeed increasing evidence that hydrophobic aggregation is correlated with toxicity for the cell [31]. Of course, the model calculations that we present here are highly simplified. The degree of hydrophobicity in real binding motifs varies, although it is typically higher than that of disordered proteins or that of the surface of globular proteins. There is, therefore, a great need for experiments to quantify the difference in aggregation behavior of signalling peptides with and without disordered flanks.

Aggregated proteins can form different structures: ordered beta sheet fibers (amyloids) or non-specific hydrophobic aggregates. Human diseases, such as Alzheimer and Parkinson disease, are mostly associated with the former. The work presented here is most closely related to the latter mechanism. Nevertheless, there is increasing evidence that the two mechanisms are connected and that hydrophobic pre-fibrillar aggregates may be causing the toxicity in amyloid forming proteins [32],[33]. Insights in (the prevention of) protein hydrophobic aggregation may therefore be important for further understanding of both aggregation types.

Of course, there could be other ways to suppress hydrophobic aggregation. For instance, aggregation would be strongly inhibited if the binding motif were embedded in a rigid structure [34]. However, a flexible binding motif has the advantage that it can combine the ability to bind reversibly with high specificity: this feature is important for regulatory motifs.

As such, it would not be surprising to find that disordered flanks have evolved to suppress aggregation. There are several other biological examples of evolutionary pressure against aggregation [34]. For example: there exist very few proteins with beta-strands on the edge of protein structures–a feature that might induce amyloid formation by edge-to-edge aggregation of beta-sheets [35]. Another example is the ‘end-capping’ of sequence regions in globular proteins that would otherwise exhibit a high amyloid-forming propensity by charged or structure-disrupting residues [36].

The stabilising effect of disordered flanks is closely related to steric stabilisation of colloids by polymers. Indeed, steric stabilization has been exploited extensively in material and drug design to stop colloids aggregating [37] or to increase the lifetime of hydrophobic drugs by attaching the drug to block copolymers with a hydrophobic middle and hydrophilic flanks [38]. The latter experiments show that steric stabilisation of hydrophobic moieties is highly relevant in biological systems but, as is often the case, evolution “discovered” this effect first.

The present work provides a testable hypothesis for the abundance of disordered regions in proteins: it suggests that disordered flanks adjacent to hydrophobic motifs can suppress aggregation of the hydrophobic peptides in solution. The hypothesis that we put forward gives a basis for in vitro or in vivo experiments into the effect of hydrophilic disordered flanks on the aggregation, solvability and toxicity of hydrophobic peptides. Confirmation of our predictions in a biological context may lead to new methods that could increase the bioavailability of hydrophobic peptides.

## Methods

### 3D Lattice Model

We use a coarse grained representation of a peptide chain where each residue occupies a single point on a cubic lattice [26]. Neighboring residues that would be covalently bound in a peptide chain are required to be on neighbouring lattice sites (Figure 1). Residues interact when residing on neighbouring sites. The internal energy of a configuration is given by:
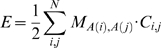
(1)where *A*(*i*) gives the amino acid at residue i, *C_i_*_,*j*_ = 1 when residues *i* and *j* interact and *C_i_*_,*j*_ = 0 otherwise. The interaction matrix *M* gives the pairwise interactions between all 20 amino acids and is based on the occurrence of amino acids in close proximity in experimentally determined protein structures [24],[25]. The interaction matrix is normalised with respect to Threonine [25], so that all pairwise interaction energies of Threonine are set to zero. We use this in our simulations to observe the purely entropic contributions of the disordered flanks.

The interaction matrix used here is based on structural proteins, while pairwise interactions in unstructured regions may have slightly different propensities. One may expect that hydrophobic residues in unstructured peptide sequence may be some what less hydrophobic due to the exposed backbone. In this case it may be that the number of hydrophobic residues needed for peptide aggregation is slightly higher than in the current work, but we expect that the qualitative effects of the aggregation remain similar.

### Monte Carlo Simulation

We use a Monte Carlo simulation technique where trial steps are accepted according to:
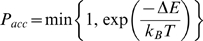
(2)where *T* is the simulation temperature, *k_b_* is the Boltzmann constant and −Δ*E* is the difference in energy between the new and old configuration of the model. Trial moves are either internal moves, changing the configuration of a chain (end move, corner flip, crank shaft, point rotation), or rigid body moves, changing the position of the chain relative to other objects (rotation, translation), see ref. [27] for more details. At each iteration a single local trial move is performed and a global trial move move (including point rotations) is performed with the probability (*P_global_* = 0.1). In the binding simulations, only rigid body moves are applied to ‘rigid’ binding motifs, whereas the configurations of the flexible binding motifs are sampled with both internal and rigid body moves.

The volume of the simulation box (60×60×60 lattice points) was kept constant, yielding a concentration for the peptide that is higher than that typical of signalling peptides in a cell (approximately 10–1000 times higher). However, the cytosol will contain other signalling peptides that, if not properly protected, could participate in aggregation. Moreover, as argued in the Supplementary Material (Text S1), the peptide solutions in our model are still sufficiently dilute to make it possible to extrapolate our findings to the typical concentrations that prevail inside a cell.

Parallel tempering, or temperature replica exchange, was used to converge more rapidly to sampling of equilibrium configurations. Multiple simulations at different temperatures were run in parallel, while trying to swap temperatures every 50000 moves with 10000 trial temperatures swaps in each simulation. A trial swap between the temperatures of two replicas was accepted with a probability [39]–[41]:
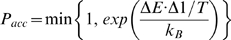
(3)


### Design of Binding Site

The design of binding interface (i.e. the contacts between the binding motif and the binding groove) was achieved through a Monte Carlo algorithm that interchanges amino acids, while optimising the total energy of the bound state and keeping the variance of the amino acids high, see [27],[28] for more details.

### Sampling of Configurations

In order to estimate the probability distribution *P*(*x*) (where *x* is an “order parameter”, such as *C_ext_*, the number of external contacts), we use both configurations of accepted and rejected trial moves weighted by the Boltzman factors of each configuration [42].

The amount of binding of the binding motif to the substrate is tracked by comparing the number of (non-covalent) contacts *C_i_*_,*j*_ in a configuration to the contacts present in the fully bound state 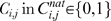. Then the total number of native binding contacts is defined as:
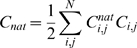
(4)where *N* is the total number of residues in the binding motif (excluding the flanking regions).

Tracking aggregation of multiple binding motifs is done by considering the total number of external contacts *C_ext_*:
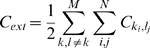
(5)where *M* is the total number of chains in the simulation box and 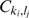 is a contact between residue *i* in chain *k* and residue *j* in chain *l*. Note that Threonine-Threonine contacts do not contribute to *C_ext_*.

The amount of binding is given by:
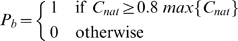
(6)The constant volume heat capacity is calculated as:
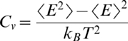
(7)Ensemble averages for an order parameter *x* are given by:
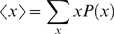
(8)where *P*(*x*) is estimated as before.

### Grand Canonical Simulation

A grand canonical Monte Carlo simulation was performed to investigate the aggregation behaviour of binding motifs at a constant (low) concentration of these peptides. Trial insertions and deletions were performed with a probability of *P_insert_* = *P_delete_* = 0.005 per move. Trial insertion of new chains (with an identical sequence) were accepted with:
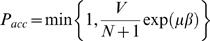
(9)and deleted with:
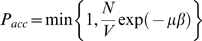
(10)where 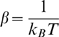, *N* is the number of free chains in the simulation box before the move, *V* is the volume of the box, and *μ* the chemical potential. The volume was kept constant at 30×30×30 lattice points and *exp*(*μβ*)was kept constant at 3·10^−6^ in all simulations. A single peptide chain was simulated in a separate box, at the same temperature, to generate new configurations for insertion into the main simulation box. Only free chains were inserted and removed, i.e. no chains that make an external contact with another chain.

Since the chains were simulated at very low density, moves are likely that remove the only peptide chain from the simulation box. At such an event the number of trial insertion moves (*M_i_*) to re-entrance was taken as:
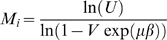
(11)where U is a random, uniformly distributed variable on the interval [0,1].

The total number of sampling steps is given by the total number of trial moves (*S*):
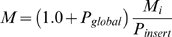
(12)The order parameters and internal energy are all zero for the empty simulation box.

### Images

Images in Figures 1 and 4 were produced using the UCSF Chimera package [43].

## Supporting Information

Figure S1 Binding motifs embedded in different environments bound to the same substrate From left to right: (A) a binding motif, (B) a binding embedded in disordered flanks and (C) a binding motif in a rigid structure. The yellow residues are hydrophobic, the blue negatively charged, the red positively charged and the grey hydrophilic.
(0.15 MB PNG)

Figure S2 Unbound binding motifs From left to right: (A) a binding motif, (B) a binding embedded in disordered flanks and (C) a binding motif in a rigid structure. The yellow residues are hydrophobic, the blue negatively charged, the red positively charged and the grey hydrophilic.
(0.06 MB PNG)

Text S1 (0.09 MB PDF)
