# Supplementary figures and images for: Disordered Flanks Prevent Peptide Aggregation
Source: PLoS Comput Biol. 2008 Dec 19;4(12):e1000241. doi: 10.1371/journal.pcbi.1000241 (PMC2588114; doi:10.1371/journal.pcbi.1000241)

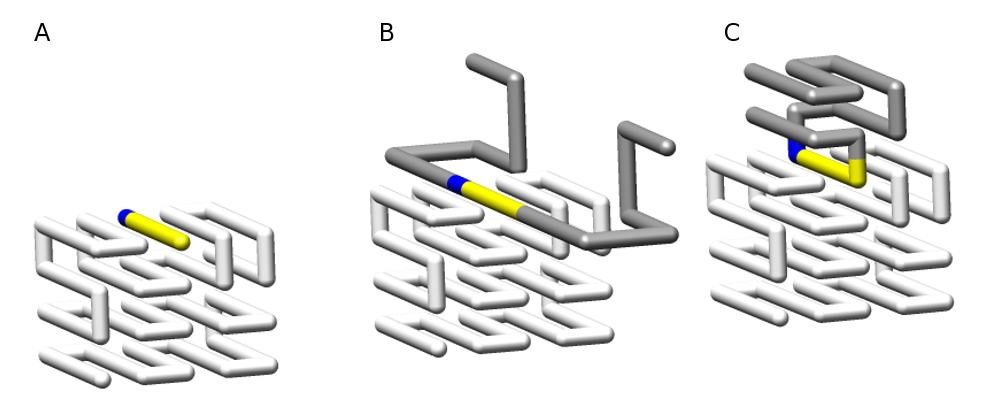

Supplement: Figure S1 — Binding motifs embedded in different environments bound to the same substrate From left to right: (A) a binding motif, (B) a binding embedded in disordered flanks and (C) a binding motif in a rigid structure. The yellow residues are hydrophobic, the blue negatively charged, the red positively charged and the grey hydrophilic. (0.15 MB PNG) [file pcbi.1000241.s001.png]

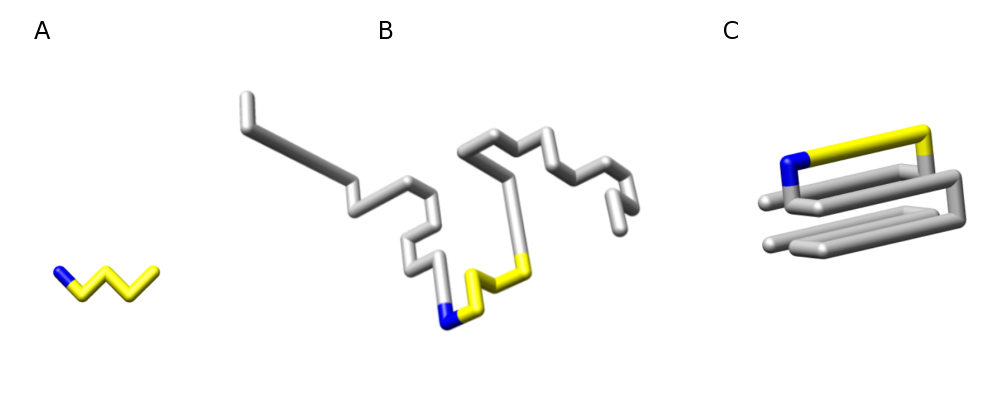

Supplement: Figure S2 — Unbound binding motifs From left to right: (A) a binding motif, (B) a binding embedded in disordered flanks and (C) a binding motif in a rigid structure. The yellow residues are hydrophobic, the blue negatively charged, the red positively charged and the grey hydrophilic. (0.06 MB PNG) [file pcbi.1000241.s002.png]
