# Supplementary material for: Disordered Flanks Prevent Peptide Aggregation
Source: PLoS Comput Biol. 2008 Dec 19;4(12):e1000241. doi: 10.1371/journal.pcbi.1000241 (PMC2588114; doi:10.1371/journal.pcbi.1000241)
Supplement: Text S1 — (0.09 MB PDF) [file pcbi.1000241.s003.pdf]

# Supplementary Material: Disordered flanks prevent peptide aggregation

Sanne Abeln and Daan Frenkel *

October 20, 2008

*FOM Institute for Atomic and Molecular Physics, Kruislaan 407, 1098 SJ Amsterdam, The Netherlands.

## Concentration dependence

To make a projection about the binding and aggregation of peptides at lower concentrations, we considered the temperature at which the aggregates (or micelles) melt and at which the peptides reversibly bind to the substrate for different concentrations. For the Grand Canonical simulations, we varied the concentration of the ideal gas reservoir from which the peptides are inserted into the simulation box and recorded the melting temperatures ($T_s$) of the aggregates or micelles as before. To estimate the temperature range for reversible binding to the substrate at low concentrations from simulations at a higher concentration, we made use of the fact that in all cases the system is sufficiently dilute to justify the use of a Langmuir relation for the fraction of bound peptides:

$$X_{\mathrm{b}} = \frac{f \cdot Q_{\mathrm{b}}/Q_{\mathrm{f}}}{1 + f \cdot Q_{\mathrm{b}}/Q_{\mathrm{f}}} \quad (1)$$

The fraction of peptides bound to the substrate ($X_{\mathrm{b}}$) may be calculated from the fraction of states with bound and unbound peptides ($Q_{\mathrm{b}}/Q_{\mathrm{f}}$). These fractions may be obtained from simulations at different temperatures. Here $f$ denotes the fugacity of the peptides; loosely speaking, the fugacity may be interpreted as the concentration of a hypothetical ideal solution of peptides at the a chemical potential $\mu = kT \ln f$. Using Equation 1 we estimated at which fugacity half of the peptides would be bound to the substrate for a given temperature ($T_b$).

Table 1 shows the approximate binding and melting temperatures for different concentrations. The reciprocal melting temperature ($1/T_s$) and the reciprocal unbinding temperature ($1/T_b$) vary roughly as the log of the fugacity. This is to be expected if both quantities follow Arrhenius behaviour. Note that in point of fact one would need a stronger binding potential energy to compensate the lower concentrations if the reversible-binding temperature-range is kept constant.

As the average interaction energy ($E_b$) decreases the change in the reciprocal temperature with respect to a constant change in fugacity becomes larger (Table 1). This indicates that at very low concentrations the peptides without disordered regions would not aggregate at the reversible binding temperature. What happens under physiological condition depends on the details of the intermolecular interactions between peptides. We hypothesize that the evolution of peptides with disordered flanks has tuned the relative interactions such that aggregation does not occur for normal concentrations. However, if for any reason the concentration of peptides without disordered flanks becomes anomalously large *irreversible* aggregation may occur.

| | bound to substrate | | aggregates | micelles |
|---|---|---|---|---|
| | BM | BM disorder | BM | BM disorder |
| $f$ | $T_b$ $(1/T_b)$ [1] | $T_b$ $(1/T_b)$ [1] | $T_s$ $(1/T_s)$ [2] | $T_s$ $(1/T_s)$ [3] |
| $3.0 \times 10^{-6}$ | 0.32 (3.1) | 0.30 (3.3) | 0.33 (3.0) | 0.19 (5.2) |
| $5.0 \times 10^{-7}$ | 0.28 (3.5) | 0.27 (3.7) | 0.28 (3.5) | 0.17 (5.8) |
| $8.3 \times 10^{-8}$ | 0.26 (3.9) | 0.25 (4.0) | 0.25 (4.0) | 0.16 (6.4) |
| $E_b$ | 6.4 [4] | 6.4 [4] | 4.9 [5] | 3.4 [6] |

Table 1: Concentration and Melting Temperatures. For each concentration the unbinding and melting temperatures are given for the binding motifs (BM) and binding motifs with disordered flanks (BM disorder). The concentration is given in the number of peptides per lattice site and the reciprocal binding and melting temperatures are given between brackets.

1 Temperature at which half of the binding motifs are bound to the substrate calculated using Equation 1; here a peptide is defined to be bound if it makes at least one contact with the substrate.
2 Temperature at which the three largest aggregated clusters melt.
3 Temperature at which the micelles melt.
4 Maximal interaction energy for peptide bound to the substrate.
5 Average interaction energy per peptide for three largest aggregates.
6 Average interaction energy per peptide for micelles with 12 peptides
